# An empirical study on the relationship between rider recreational specialization, individual-environment fit, and heart flow experience

**DOI:** 10.1016/j.heliyon.2024.e31781

**Published:** 2024-05-23

**Authors:** Wei Ye

**Affiliations:** College of Physical Education, Henan University of Science and Technology, Luoyang, China

**Keywords:** Recreational specialization, Individual-environment fit, Heart flow experience, Pleasure

## Abstract

The aim of the study is to create and validate a model of the relationship between specialization in leisure activities, the individual's adaptation to the environment and the heart flow experience. In order to clarify the role of the individual's adaptation to the environment in the relationship between specialization in leisure activities and the heart flow experience. The study utilized purposive sampling, cluster sampling, and random sampling. Using questionnaires and interviews to survey 525 cycling enthusiasts. Descriptive analysis, model construction and testing of the constructed path relationships were conducted using SPSS 20.0 and Amos 20.0. The results indicate that the model of the relationship between recovery specialization, individual-environment fit and heart flow experience has a good overall fit. The model shows good reliability and validity. Cyclists' recreational specialization has a statistically significant effect on individual-environment fit (*β* = 0.38, *P* < 0.001). The fit between individual and environment has a statistically significant influence on the heart flow experience (*β* = 0.39, *P* < 0.001). The fit between individual and environment serves as a mediating variable between recreational specialization and the heart flow experience, with the path showing statistical significance (*β* = 0.15, *P* < 0.001). Recreational specialization has a statistically significant effect on the heart flow experience (*β* = 0.30, *P* < 0.001). And the overall path of the effects of recreational specialization of cyclists on the fit between individual and environment is (*β* = 0.45, *P* < 0.001), with the path showing statistical significance.

**Conclusion:**

The stronger the recreational specialization of cyclists and the greater the fit between individual and environment, the stronger their heart flow experience. The fit between individual and environment plays a partially mediating role.

## 1 Introduction

The development of leisure sports has led to an increasing popularity of cycling among the public. As a form of fitness, cycling not only fulfills the needs of cyclists for sightseeing and physical exercise but also provides deep leisure experiences. And it brings both physical and psychological pleasure from the journey. In the academic circle, cycling has become a focal point in the study of leisure sports. Scholars conduct in-depth research on its origins, current status, development trends, motivations, culture, characteristics, and experiences. They have achieved numerous valuable results. Particularly in the field of cycling experience research, it has become a prominent topic in psychological research and has identified many predisposing factors. However, there is relatively limited research on the degree of cycling specialization and the fit between individual and environment as predisposing factors. Therefore, exploring the impact of cyclists' specialization level and the fit between individuals and their environment on their heart flow experience represents a highly innovative approach. In leisure studies, research on individual specialization and psychological aspects primarily focuses on analyzing athletes' skills, psychological activities, and level of specialization in recreation. Research on the fit between individuals and their environment primarily focuses on the impact of heart flow experiences in leisure and tourism on satisfaction with environmental attributes. However, conclusions vary depending on different research subjects and projects, indicating further value for in-depth research. Relevant literature also suggests that heart flow experience is a psychological state in which an individual is fully immersed in a particular activity. It dynamically changes with different sports environments and individual specialization levels. Therefore, starting from the weakness of the existing literature results, the study takes cycling enthusiasts as the research object. It aims at targeting to further explore the relationship between cyclists' recreational specialization, individual-environmental fit and the structure of the mind-flow experience. Clarifying the strength of influence of antecedent factors on the cyclist's experience of heart flow. So as to further promote the participatory behaviors of the cyclists, and at the same time, to provide certain theoretical support for the leisure academics.

### 1.1 Literature review

#### 1.1.1 Theory of individual-environment fit

Individual-environment fit is a fundamental theory in the fields of management and psychology. Individual-environment fit refers to the equilibrium between the resources and needs of both an individual and their environment. This equilibrium is shaped by the interactions between an individual and their surrounding environment. A high level of fit has a positive impact on an individual [1]. Individual-environment fit can be divided into supplementary fit and complementary fit. Supplementary fit denotes the similarity between an individual and other members of an organization in terms of traits, representing the alignment among organizational members. This is evident in the mutual complementarity and compatibility between an individual's personality traits, values, goal attitudes, and organizational culture. Complementary fit refers to the degree of alignment between an individual and their organization, representing the match between organizational members and the environment. In theory, complementary fit can be categorized into demand-supply and requirement-capability fit. The former pertains to the ability of the work environment to provide what an individual needs, while the latter denotes that an individual's capabilities meet the necessary conditions for the job. The concept of complementary fit has been extended to leisure studies, defined as the compatibility between leisure seekers and their leisure environments. It describes the interaction and compatibility between recreationists and their surroundings. And it measures whether the provision of leisure environments aligns with the needs of recreationists, and whether the abilities possessed by recreationists match those of the leisure environments. When a leisure environment aligns with the expectations of recreationists, it achieves a demand-supply fit. Similarly, when a recreationist's skills align with the requirements of their surroundings, it achieves a requirement-capability fit, leading to a sense of well-being in recreation [2].

The compatibility between recreationists and the recreation environment is achieved when one of the following conditions is met: 1) at least one party fulfills the needs of the other; 2) both parties share similar fundamental characteristics; 3) both are present. The higher the compatibility between the two, the more enhanced the recreation experience and satisfaction of the recreationist.

#### 1.1.2 Theory of recreational specialization

Recreational specialization is a continuum of behaviour that progresses from general interest and low involvement to specialist interest and high involvement. It is created by learning and is reflected by the equipment, skills and site preferences used in the activity [3]. Subsequent researchers have successively proposed the inclusion of the individual's emotional attachment to the development of the activity and social benefit variables to evaluate recreational specialization. They believe that recreational specialization is a cyclic developmental process that occurs when individuals develop cognitive, behavioral and affective responses to a particular leisure activity. And that it is a combination of cognitive, behavioral and affective responses to the domain of the activity [4]. Cognitively, individuals prioritize technological advancement and knowledge acquisition. Behaviorally, they emphasize active participation. Emotionally, they place value on their identification with the activity and themselves or the attachment generated by combining leisure activities with social circles. After over 40 years of development, recreational specialization theory has been applied in various leisure activities such as mountain sports, cycling, and exploration, accompanied by numerous specialized research topics.

Some scholars have proposed four main points in their discussion of the socialization process of recreation participation among leisure activities, based on the relationship between the value of individual activities, behavioral responses, and stages of specialization development [5]: (1) Recreational experiences can be used as predictors of recreation behavior, where increased participation time leads to a higher level of individual specialization in recreation; (2) Participants with specialized characteristics have a specific group circle which is a sub-cultural group with distinctive recreational values; (3) Individuals' considerations regarding the benefits and consumption of recreation gradually shift towards a focus on the essence of the activity and its environment; (4) Enhanced specialization capabilities enable recreationists to better anticipate activities and environments, fostering a closer connection with recreational resources. The level of recreational specialization manifests in different types of participation as individuals invest more time and accumulate experience.

The specialized recreation groups are often homogeneous, with the level of specialization among participants linked to their respective group membership. The activities organized by the group influence participant behavior. For example, each group has at least one major activity, a base from which it takes place, skills and organizations that respond to the needs of the group to respond to the group's leisure activities, etc. The leisure behavior of specialized recreationists demonstrates consistency and is often characterized by spontaneous professional growth. Some scholars have categorized the social circles of specialized recreationists into low-specialization, intermediate-specialization, and high-specialization social circles, each with its own group-specific functions and community culture [6]. Additionally, some scholars have identified eight characteristics of specialized recreation [7]:

(1) As the duration of engagement in a particular recreational activity lengthens, the specialization of the participants also deepens;
(2) –(8) As specialization in recreation increases, the emotional, time, and financial investments of recreationists will also increase. The centrality of the activity in their lives will rise, leading to greater acceptance and support of associated norms and established procedures. There will be a heightened emphasis on equipment and the necessary skills for its use, as well as a greater reliance on specific resources. Attention to relevant information about the activity will also increase. Recreationists will value the experience of the activity more than the activity itself.

#### 1.1.3 The theory of heart flow experience

The heart flow experience refers to the psychological state achieved by an individual when fully immersed in an activity, characterized as a self-purposeful experience [8]. In this state, the individual becomes absorbed in the activity they are engaged in, experiencing joy and enjoyment from the process. It is characterized by a fusion of action and awareness, a high degree of concentration, a sense of control over one's actions, a perception of time distortion, and a sense of self-contained experience. This intrinsic motivation drives individuals to repeatedly engage in the activity.

The heart flow experience model has undergone three evolutions. According to their progressive stages, it can be categorized as the three-dimensional heart flow model, the four-dimensional heart flow model, and the eight-dimensional heart flow model. The aim is to delineate individuals' perceptions and outcomes in various scenarios regarding the conjunction of challenges and skills.

The three-dimensional heart flow model primarily examines the relationship between skills and challenges. When a participant's skills surpass the level of challenge, boredom may arise. Conversely, when an individual's skills are insufficient for the challenges at hand, anxiety may result. Only when there is a balance between challenges and skills can the participant enter a state of heart flow experience quickly. However, as the challenges evolve, there may be a temporary mismatch between the challenges and the skills, resulting in feelings of boredom or anxiety (for example, when an individual's skills are not suited to the complex activity environment, anxiety may resurface). Therefore, according to this model, an individual's skills must correspond with their perceived cognitive challenges in order to achieve a state of heart flow. But the balance between challenges and skills is determined subjectively by the individual rather than by objective criteria [9,10].

With the advancement of heart flow experience theory, some scholars have proposed a four-dimensional flow model based on extensive empirical research. This model suggests that an individual's heart flow experience occurs only when both skills and challenges surpass a certain threshold. Furthermore, they have integrated the concept that boredom arises when participants perceive challenges and skill levels to be lower than their usual activity experience into the four-dimensional heart flow model [11]. The revisions signify a significant advancement in the conceptualization and methodology of heart flow theory.

The four-dimensional heart flow model is primarily constructed from activities characterized by extreme complexity and engagement, such as rafting, cycling, and mountain exploration. However, everyday activities may only periodically meet such criteria. Additionally, some scholars argue that the four-dimensional heart flow model may not sufficiently capture participants' heart flow experiences as the balance between challenge and skill level fluctuates [12]. As a result, in order to enhance the applicability of heart flow experiences, some researchers have expanded the original four-dimensional flow model into an eight-dimensional model. This revised model categorizes challenge and skill levels into high, medium, and low tiers, as opposed to purely high and low levels. Additionally, within each tier, four additional dimensions are introduced: stimulation under high challenge and moderate skill, relaxation under low challenge and moderate skill, mastery under moderate challenge and high skill, and apprehension under moderate challenge and low skill. It is essential to note that heart flow experiences only occur in situations with both strong challenge and elevated skill levels [13]. The eight-dimensional flow model encompasses stimulation, flow, mastery, boredom, ease, indifference, apprehension, and anxiety. However, when the difficulty of the challenge is too high, if the participant continues to engage in the activity, with the gradual improvement of skills, his experience will be from worry, anxiety, excitement to the eventual generation of the flow of the heart. However, although this model is more able to explain the psychological feelings of the participants, the structure is still not separated from the four-dimensional model of the flow of the heart.

### 1.2 Research inference

#### 1.2.1 The impact of recreational specialization on individual-environment fit

The literature on recreational specialization indicates that recreationists have distinct preferences for their activity settings. For example, researchers suggest that the level of specialization among cyclists influences their preferences for activity venues. Qualitative research has shown that highly specialized cyclists possess extensive tacit knowledge and greater control over the cycling environment, leading them to prefer routes and paths that align with their preferences [14]. This study aligns with Tian's findings on senior cycling enthusiasts who tend to prefer remote routes, indicating that these individuals' preferences contribute to their sense of self-identification and result in more positive emotional experiences. Additionally, these preferences foster high motivation and willingness to exert greater effort in completing tasks [15]. Researchers studying rock climbers have also confirmed this information. Recreational specialization of rock climbers is positively related to individual-environment fit. Their findings suggest that climbers with different specialization characteristics seek distinct environmental attributes [16]. Some scholars argue that factors such as gender, level of specialization among participants, leisure motivations, and perceptions of mountain environments do not affect the difficulty and novelty of mountain route choices (e.g., route, technicality, convenience, wilderness, difficulty, safety). Their aim is to pursue the joy and wonder of mountain exploration [17]. The research findings from these two studies may vary due to differences in the characteristics of the research subjects. The level of recreational specialization among cyclists varies, leading to diverse environmental preferences. Individuals with a high degree of specialization tend to prefer routes with fewer man-made facilities, while those with lower specialization levels prefer accessible routes. However, the enjoyment of cycling is a common pursuit shared by all cyclists [18]. Yueh (2020) suggests that cyclists with higher levels of recreational specialization tend to prefer routes with greater difficulty and novelty, while those with lower specialization levels are more inclined towards convenient activity bases, artificial facilities, and a high safety factor [19]. Zhang and Wu (2014) propose that leisure behavior is deliberate and goal-oriented [20]. The scholars above have confirmed that the degree of recreational specialization among individuals has an impact on their compatibility with the environment.

#### 1.2.2 The influence of individual-environment fit on heart flow experience

The selection of leisure exercise environments by individuals is influenced by three factors. These three factors are derived from “cultural predispositions” in the sense of “meaning”, the individual's assessment of their own conditions, and their evaluation of the leisure exercise environment. The pursuit of a fit between the leisure exercise environment and their own benefits drives the individual's “cultural orientation” in the sense of “meaning,” aiming to achieve an optimal match between supply and demand. The individual's self-assessment of their own conditions reflects their evaluation of their abilities, resources, cultural and social capital in engaging in leisure exercise. Evaluate whether they are able to fulfil their sporting aspirations through participation in recreational sports programs. The higher an individual's assessment of their conditions, the stronger their belief in leisure exercise becomes. The purpose of this assessment is to estimate the degree of fit between the individual and the leisure exercise environment. Higher fit facilitates a more effortless experience of positive emotions such as relaxation, a sense of life meaning, and heart flow experiences during leisure exercise.

The integration of personal traits, needs, leisure activities, and the environment among active individuals can enhance their satisfaction, heart flow experience, and well-being. Some scholars have observed that regular walkers prefer to walk on sidewalks with better greenery during the day through research. They suggest that optimal environmental attributes attract them to walk, leading to a sense of leisurely enjoyment. The degree of compatibility between personal skills and the challenges posed by the recreation environment is implicit in the level of heart flow experience [21]. Some scholars have classified cyclists into four groups based on their heart flow experience: ordinary, experience fit, fit, and perfect fit. The levels of heart flow experience corresponding to each group increase sequentially [22]. Some scholars have attributed the participants' focus, control, pleasure, and sense of time distortion to the environmental resources provided by the activity venue, social opportunities, environmental functions, facilities, and management [23]. Furthermore, it is evident that the alignment between an individual and their environment can be used to predict the heart flow experience of an individual during an activity.

#### 1.2.3 The relationship between recreational specialization and heart flow experience

The expertise and knowledge of the cyclist are the primary factors that can predict their heart flow experience. Participants can experience the pleasure of physical activity and recognize the psychological and physiological advantages of cycling. Some scholars, who have focused on mountain climbers as their research subjects, propose that as a mountain climber's level of recreational specialization increases, so does the likelihood of experiencing flow. They achieve higher scores in terms of challenge level, and heart flow experiences also inspire individuals to seek new challenges in order to improve their skills and elevate their level of specialization [24].

The emotions, cognition, and actions of individuals towards leisure activities can impact their level of recreational specialization. Specialization is a gradual process with no defined limit, similar to the concept of limitless heart flow experiences. Skill and challenge are key factors in measuring both specialization and heart flow experiences. The level of specialization is evident in the participants' skills, equipment used, and preferred environment. Heart flow experiences are not constant and can be influenced by various factors. If participants are limited by their own skills and cannot achieve a balance with challenges, their heart flow experience may be weak or non-existent [25]. Participants with a higher level of specialization may experience enhanced heart flow experiences, reaching an optimal point when their personal skills and challenges are in balance. The emotional, cognitive, and behavioral aspects of specialization in athletes show a significant positive correlation with dimensions of the heart flow experience, and can predict levels of focus, time distortion, integration of consciousness with movement, and behavioral experience. Questionnaire surveys conducted on cyclists by numerous scholars confirm that recreational specialization is positively associated with leisure benefits and heart flow experiences. The greater the degree of recreational specialization among cyclists, the stronger their feelings of heart flow experiences [26].

The existing literature lacks thorough exploration of the mediating role of individual-environment fit in discussions. Kelly et al. (2010) suggests that a higher degree of recreational specialization among drifters and a better fit between their recreation environment lead to a stronger heart flow experience. They also confirm that recreational specialization and individual-environment fit are antecedent variables of heart flow experience, with individual-environment fit serving as a mediating variable. It further demonstrates the moderating effect at the level of environmental functional fit [27]. However, some researchers have concluded from their study on college students' orienteering activities that the sports environment is a significant factor hindering the generation of flow states among college students, although it does not act as a mediating factor [28]. The research findings may be attributed to unfamiliar sports environments that do not support the performance improvement of college students, leading to a decrease in their heart flow experiences. Therefore, this study focuses on cyclists as the research subjects to further explore whether there is a mediating effect of individual-environment fit.

### 1.3 Research inference

Based on theoretical reasoning (1.2 Research inference), the following research hypotheses are proposed:

H1 The influence of recreational specialization on individual-environment fit is statistically significant.

H2 The influence of individual-environment fit on heart flow experience is statistically significant.

H3 The influence of recreational specialization on heart flow experience is statistically significant, and individual-environment fit plays a mediating role.

## 2 Research design

### 2.1 Demographic characteristics

The study examines the impact of cyclist specialization and individual-environment fit on heart flow experiences, focusing on the relationship between cyclists established through WeChat and in-person engagement to convey the research content. Respondents were selected from 19 cycling groups in Henan Province, each with a minimum of 20 members. All participants had at least 6 months of cycling experience, possessed verbal communication and athletic abilities, and signed written informed consent. Primary data were collected through on-site observation, interviews, and participation as quantitative analysis materials.

Interviews and observations: Follow, listen, observe and interview during the rest time, the gathering time and the network chat time during the riding.

A method of on-site whole-group sampling research was employed for participants in cycling, while non-on-site cyclists were randomly sampled using WeChat groups as the platform. The questionnaire was completed and collected within a limited time frame through online research.

Over a period of four months, a total of 651 questionnaires were distributed, out of which 592 were returned. After excluding invalid questionnaires (due to missing answers), 525 valid questionnaires were obtained, resulting in an effective response rate of 88.68 %.

### 2.2 Research tools and their reliability and validity assessments

The research on variable indicators drew upon indicators from academic journals and published literature. Under the guidance of professional instructors, the study was finalized after integrating data from initial in-depth interviews with 36 experienced cycling enthusiasts. Subsequently, the scientific validity of the content was ensured through soliciting opinions from six experts in related research fields.

Based on Zheng et al. (2005)."Cycling Recreational Specialization Scale” [29], the research scale was modified. The recreational specialization scale consists of 15 item factors, organized into three levels: emotional, behavioral, and cognitive. The behavioral level includes 6 item factors, the cognitive level consists of 5 factors, and the behavioral level includes 4 item factors. The Cronbach's a value is 0.923, and the KMO value is 0.933, indicating strong reliability of the scale.

Based on Chen et al.(2019) research questionnaire [30], we have adapted it to develop the "Cyclist's Individual-Environment Fit Scale” for this study. This scale consists of six domains, namely, environmental resource fit, social fit, environmental function fit, environmental facility fit, activity equipment fit, and capability-requirement fit. Each domain includes two, three, four, two, two, and four item factors, respectively, totaling 17 item factors. The scale demonstrates high reliability with a Cronbach's alpha value of 0.931 and a KMO value of 0.920.

Based on Qu's (2013) research questionnaire [31], we have adapted it to develop the “Rider Heart Flow Experience Scale” for this study. The scale consists of 15 items, categorized into seven domains, namely, sense of control, focus, perception of time distortion, integration of consciousness with movement, clear goals, behavioral experience, and perceived challenge. The behavioral experience domain comprises three items, while the remaining six domains contain two each. With a Cronbach's alpha value of 0.928 and a KMO value of 0.901, the scale demonstrates high reliability.

The scales above are assessed using the Likert-5 scale, with scores ranging from 1 (strongly disagree) to 5 (strongly agree). A higher score indicates a greater level of recreational specialization, individual-environment fit, and heart flow experience among individuals.

### 2.3 Mathematical statistics

Descriptive and factor exploratory analyses were performed using SPSS20.0 software, followed by factor fitting analysis using Amos20.0 software. Subsequently, a structural equation model was employed to examine the relationship between recreational specialization, individual-environment fit, and heart flow experience among cyclists. All tests were performed with bilateral α-level of <0.05 to indicate statistical significance.

## 3 Results

### 3.1 Demographic characteristics of cyclists

The demographic characteristics of cyclists are outlined in Table 1, indicating a predominantly male population (70.3 %) with the majority aged over 31 (84.6 %). Furthermore, more than half of the cyclists have attained junior college education or below (52.8 %). The most common cycling duration among cyclists is 1–2 years, accounting for 47.2 % of the population.

**Table 1:** Demographic characteristics of cyclists.

Population situation	Factor	Number of individuals (%)	Population situation	Factor	Number of individuals (%)
Gender	Male	369(70.3 %)	Educational level	Junior college education or below	277(52.8 %)
	Female	156(29.7 %)		Bachelor's degree or above is required	248(47.2 %)
Age (in years)	≤30	81(15.4 %)	Cycling duration (years)	0.6–1	103(19.6 %)
	31∼45	201(38.3 %)		1–2(inclusive)	284(54.1 %)
	≥46	243(46.3 %)		>2	138(26.3 %)

### 3.2 Model fitting and parameter estimation testing

Utilizing statistical software to conduct descriptive, reliability, validity, factor analysis, and model exploration analyses on the data from 525 cyclists. In addition, employing interview methods and a literature review to validate the quantitative data.

Table 2: The scale underwent an Aggregate Validity Test and Principal Component Analysis using SPSS 20.0 software. The cumulative proportion of variance accounted for by the extracted common factors ranges from 72.814 % to 85.329 %, exceeding the standardized value of 50 %. When the absolute value of the skewness coefficient exceeds 2, and the absolute value of the kurtosis coefficient exceeds 5, it is considered abnormal. In this study, the kurtosis values range from 0.018 to 0.869, and the skewness values range from 0.180 to 0.811, adhering to statistical criteria. Following Principal Component Analysis, all items demonstrate factor loading between 0.762 and 0.855, surpassing the standardized value of 0.5, indicating that the scale exhibits good Aggregate Validity.

**Table 2:** Summary of aggregate reliability testing results for the scale.

Scale	Accumulated interpretation Variability	Kurtosis	Skewness	Factor load
Specialization in Recreation	72.814 %	0.018–0.864	0.397–0.689	0.762–0.836
The fit between an individual and their environment	79.471 %	0.199–0.869	0.180–0.552	0.762–0.855
Heart flow experience	85.329 %	0.043–0.544	0.516–0.811	0.770–0.855

Regarding structural validity, an acceptable range for χ2/df is 1–3, with lower values being preferable. The NFI, GFI, AGFI, IFI, RFI, and CFI values are generally expected to exceed 0.9, with higher values indicating better fit. PGFI, PNFI, and PCFI values typically should surpass 0.5, with larger values being more desirable. The RMSEA value is generally expected to be less than 0.1 for a excellent fit, while the RMR value should ideally be less than 0.05. As shown in Table 3, the model's fit indices suggest a strong level of construct validity in this study.

**Table 3:** Summary of fitting degree.

χ2/df	NFI	GFI	RMSEA	RMR	AGFI	GFI	IFI	RFI	CFI	PGFI	PNFI	PCFI
1.171	0.966	0.972	0.018	0.02	0.962	0.972	0.995	0.959	0.995	0.722	0.813	0.837

### 3.3 Factor testing

The unstandardized regression coefficients for the 47 factors in the three research questionnaires were estimated using the maximum likelihood method. The C.R. values for all factor levels ranged from 7.763 to 14.286, with P < 0.001, and S.E. values between 0.020 and 0.041 were observed. There were no negative errors of external variables, and the model satisfied essential fit testing criteria. The average variance extracted for the factors of recreational specialization, individual-environment fit, and heart flow experience questionnaires were approximately 0.4950, 0.5074, and 0.5187 respectively, while composite reliabilities met statistical standards at values of 0.7461, 0.8605, and 0 0.8829 respectively.

### 3.4 Path relation analysis of the model

**Fig. 1:**
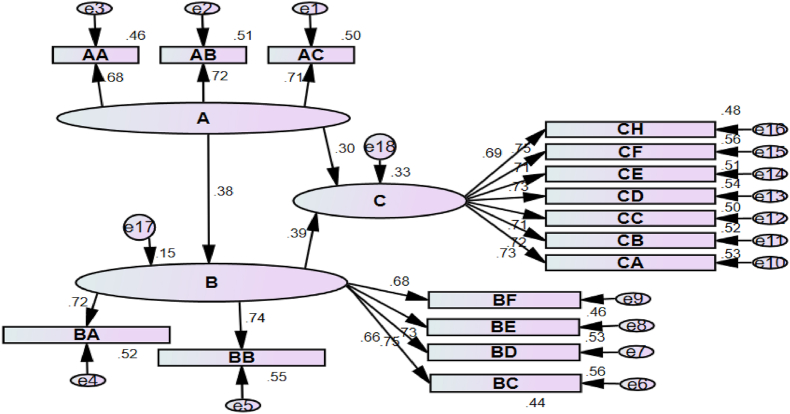
Structural equation model Note: recreational specialization (A): cognition (AA), emotion (AC), behavior (AB). Individual environment fit (B): ability and requirement fit (BA), environmental function fit (BB), activity equipment fit (BC), social fit (BD), environmental resource fit (be), and environmental facility fit (BF). Heart flow experience (C): sense of control (CA), focus (CB), behavior experience (CC), integration of consciousness and movement (CD), sense of time warp (CE), clear goal (CF), clear understanding of challenge (CH).

Based on Fig. 1 and Table 4, it is apparent that the level of recreational specialization among cyclists has a significant and direct positive influence on the individual-environment fit (*β* = 0.38). A higher degree of recreational specialization among cyclists corresponds to a stronger preference for individual-environment fit. This indicates research hypothesis 1 is valid. The positive impact of individual-environment fit on heart flow experience (*β* = 0.39) suggests that cyclists with a higher degree of fit tend to have stronger heart flow experiences. This supports hypothesis 2. The level of recreational specialization among cyclists has a direct and significant positive impact on their heart flow experience (*β* = 0.30). A higher degree of recreational specialization is associated with a stronger sense of heart flow experience during cycling. Additionally, the study found that the level of recreational specialization indirectly influences heart flow experience through the mediating variable of individual-environment fit (*β* = 0.15). The higher the degree of recreational specialization among cyclists, the greater their expectation of achieving a fit between their skills, experience, and the environment, thereby enhancing their heart flow experience. Path fusion reveals a significant total path relationship of 0.45 between the degree of recreational specialization among cyclists and heart flow experience, providing support for hypothesis 3.

**Table 4:** Summary of path coefficients.

Hypotheses	Path	Direct effects	Estimate	C.R.	Indirect effects	Total effect	Decision
H1	A→B	0.38***	0.391	6.399			Supported
H2	B→C	0.39***	0.440	7.063			Supported
H3	A→C	0.30***	0.349	5.285	0.15***	0.45***	Supported

Note: ***P < 0.001; **P < 0.01; *P < 0.05.

## 4 Discussion

### 4.1 An analysis of the impact of riders' specialization in recreation on individual-environment fit

Humanists proposed that the challenge of exercise transforms individuals into agents of movement, thereby emphasizing the significance of the exerciser's presence. Throughout training, the exerciser also discovers tranquility and pleasure in their psyche. The implication is that the difficulty of exercise arises from the alignment between the exerciser's capabilities and the environment in which they participate. Additionally, the exerciser assesses their own exercise experiences, and these assessments can drive corresponding exercise intentions, further shaping their future behavior [32]. Based on this theory in conjunction with the data from the interviews, it is clear that cyclists, depending on their degree of specialization in recreation, prefer environments that suit their preferences and aim to maximize the benefits of cycling. Furthermore, the cyclists surveyed are predominantly riding in groups, and an environment tailored to the individual facilitates interaction between cyclists. Cycling tends to be fun and this atmosphere is preferred by cyclists. Numerous findings from the literature are consistent with the results of this study. Cyclists with high self-efficacy tend to engage for longer periods of time and focus more on their own skills, riding ability and adaptation to the cycling environment as their cycling experience increases, with the aim of developing a sense of the benefits of recreational cycling [33].

In-depth interviews with several experienced cyclists revealed that they categorize cyclists according to the frequency of their participation, their technical ability and their location preference. This categorization is divided into four groups: infrequent cyclists, general cyclists, specialized cyclists in terms of riding technique, and specialized cyclists in terms of riding location. The distinction between the first three groups is based on differences in experience, technical ability and commitment to cycling. As cyclists become more specialized, they gradually become masters of preferred resources and evolve into specialized cyclists in terms of riding technique and location. It is also evident from the interview transcripts of the cyclists that as they become more specialized, their experience and skills gradually improve. Cyclists with higher levels of specialization tend to favor challenging routes with varied road conditions, while those with lower levels of expertise opt for easier routes. This indicates that as cyclists continue their engagement in the activity over time, they seek a desired alignment between individual abilities and environmental compatibility. This concept has been supported by relevant literature [34]. Tang et al.(2014) discovered that mountain climbers with a high level of recreational specialization also exhibit a stronger sense of place attachment. Additionally, the past mountaineering experiences of climbers serve as a precursor to place attachment, establishing it as a prerequisite for revisiting a location due to psychological attachment [35]. Some scholars propose that the higher the level of recreational specialization among recreationists, the stronger their intensity of place attachment [36]. Based on this, it is apparent that cyclists selectively choose cycling environments based on their level of recreational specialization. They opt for routes that cater to their needs and preferences in order to experience the positive and affirming perception that such environments provide for cyclists.

### 4.2 Analysis of the impact of individual-environment fit on heart flow experience

The heart flow experience experienced by cyclists is characterized by four significant elements. The cycling environment aligns with the cyclist's expectations. The cyclist is fully engaged in the ride and finds enjoyment in the activity itself. There exists a balance between the challenges of cycling and personal skill. And the cyclist experiences a sense of control over their ride. Therefore, in the heart flow model for cyclists, both the objective environmental factors of the ride and the cyclist's skill level are essential. If the cycling environment does not align with the individual's desired goals or if it is not compatible with the cyclist's abilities, it becomes more challenging for them to achieve a state of heart flow. When the objective environment of the ride provides natural resources or facilities that meet the cyclist's desired needs, there is a higher level of compatibility between the cyclist and their surroundings. In such cases, the cyclist becomes fully engaged in the activity, utilizing their skills to effectively manage and control their ride while immersing themselves in a state of heart flow.

The qualitative interviews in this study yielded that a supportive cycling environment and positive relationships among cyclists, combined with feedback, can enhance the individual's psychological well-being. Conversely, an unsupportive cycling environment or distant relationships among cyclists may diminish the cyclist's experience and lead to feelings of boredom or anger. Therefore, if a cyclist perceives a positive atmosphere among their companions during the journey (including encouragement, harmony, affirmation, and effort). This positive atmosphere represents social fit and can facilitate deep involvement in cycling. This conclusion aligns with Brown's(2016) research but differs in certain aspects [37]. When the cycling environment aligns with the individual's specific conditions (e.g., fit of environmental resources, functional fit of the environment, availability of facilities, suitability of activity equipment, and alignment with ability requirements), the cyclist is more likely to experience a state of heart flow. According to interviews conducted in this study, when the cycling environment meets the cyclist's personal expectations, they become fully engaged in the cycling activity, feeling a sense of control over their situation and having a clear goal in mind. This leads to a merging of consciousness and cycling experience amidst an altered perception of time, fully immersed in the fluidity of cycling [38]. Furthermore, a cycling environment devoid of complex distractions can elicit more positive emotions compared to the routine of daily life at home or work. The study delves into the positive sentiments experienced by cyclists, which primarily include feelings of novelty, enjoyment, surprise, relaxation, challenge, and confidence. Some scholars propose that the positive emotions associated with cycling are largely influenced by individual perception, skill level, and compatibility with the road environment. These research findings align with those of Lin et al.(2017). He suggested that outdoor activities generate positive emotions and provide participants with fulfilling experiences such as purpose and meaning, personal growth, achievement, and self-acceptance [39]. Through in-depth interviews with cyclists, the study identified four types of realizations as components of the cyclists' heart flow experience: “dynamic integration of skills and road conditions”, “sense of motor control”, “satisfaction with the motor journey” and “identity building”. Four types of realizations were identified as components of the cyclists' experience of heart flow.

The primary motivations for cyclists to pursue the activity of cycling are the social and health benefits. Interviews with 205 cyclists revealed that they all engage in cycling as a collective group, seeking joy and exploring meaning in life to achieve personal growth. Additionally, through their participation in cycling, they experience a refreshing feeling, rejuvenate their spirit, and pursue health. This discovery aligns with the findings of Sun and colleagues(2024), who propose that when an individual's interaction with the environment aligns with tourists' expectations, it can lead to a sense of engagement due to the potential for experiences such as fitness, stress relief, and pleasure in tourism. These experiences cater to tourists' needs and serve as a critical motivation for promoting the further development of individual recreational specialization [40].

The heart flow experience is also influenced by the alignment of an individual's skills with their athletic environment. This study further validates previous research suggesting that personal athletic abilities contribute to a sense of mastery over the environment or activity, allowing individuals to effectively navigate challenges in the athletic context and promoting the occurrence of heart flow. Additionally, heart flow represents an optimal internal experience characterized by deep engagement in a suitable athletic environment and a high level of enjoyment resulting from motivated participation. This concept is akin to the theory of person-environment fit. As a result, when cyclists' riding skills are more closely aligned with the environment in which they ride, they become more fully engaged in the activity, absorbed in the integration of self-awareness and movement, and savoring the cycling experience. They perceive time as passing quickly during their ride, achieving an optimal psychological state for cycling.

Furthermore, research has also shown that the alignment between an individual and their environment, as well as their sense of competence, play a role in balancing skills and challenges, ultimately enhancing the athlete's movement experience [41]. As the relationship between challenges and skills evolves, an individual's heart flow experience will also fluctuate. With increasing challenge difficulty, individuals must acquire new skills, while improving skill levels prompt them to seek higher-difficulty challenges in order to achieve a smooth heart flow experience. Applying this argument to cycling underscores the critical role of skill and challenge in triggering a cyclist's heart flow experience. An individual's experience of flow is also influenced by the interaction between their skills and the challenges presented by the cycling environment, indicating that the fit between an individual and their environment reflects the extent to which their skill level aligns with and meets the demands of the cycling environment.

### 4.3 An analysis of the impact of recreational specialization on heart flow experience

Based on the interview data, it is evident that cyclists with a deeper involvement in cycling possess more experience and greater compatibility between their skills and the cycling environment, making them more likely to experience a joyful flow during their journey. These heart flow experiences also lead cyclists to become more focused on the activity. This finding aligns with the argument that expertise in an activity is highly correlated with the attributes of the activity environment. Some scholars have studied bicycle riders and concluded that riders' recreational specialization can effectively predict their heart flow experience, with skill and knowledge levels having the highest predictive power [42]. This suggests that an individual's level of recreational specialization can impact their experience of flow. Additionally, a study involving 136 online game players found that experienced players are more likely to experience flow and display addictive tendencies compared to inexperienced players. Longer online sessions were also linked to a deeper sense of immersion, increasing the likelihood of developing an online addiction [43]. The conclusion aligns with our research findings that indicate a positive correlation between the level of recreational specialization among cyclists and their intensity of heart flow experience. Despite differing research fields, the arguments presented are congruent.

The phenomenon of recreational specialization among cyclists can be attributed to the theory of deep leisure. This phenomenon is characterized by a group of enthusiasts or individuals who willingly participate in organized cycling activities. For these participants, the opportunity to demonstrate their unique athletic abilities and related knowledge through cycling brings them pleasure, serving as a motivation for engaging in high-involvement behaviors and experiencing a sense of flow during cycling. The recreational specialization of cyclists encompasses elements such as leisure experience, skill, knowledge, sharing pleasure with partners, and centrality in life. Therefore, the theory of recreational specialization among cyclists is typically based on emotions, cognition, and behavior and involves a high degree of emotional attachment and intrinsic loyalty. The commitment to cycling continually reinforces the individual's loyalty to cycling and the pursuit of desired athletic benefits. Some scholars have proposed that the psychological state of long-distance cyclists during smooth cycling includes feelings of physical and mental ease, excitement, self-actualization, increased speed, freedom, slower movements, a sense of integration with the bike, and satisfaction [44].

The causal relationship between recreational specialization and the experience of heart flow can be attributed to the degree of involvement of cyclists' actions and awareness. When an individual views cycling as a fundamental and personally significant focal point of their life, their involvement in cycling will intensify. During the process of engaging in physical exercise, individuals may undergo psychological states such as exhilaration, absorption, and concentration. Additionally, findings from a literature review suggest that when athletes achieve a balance between their skill level and the challenge presented by their sport, or even surpass these challenges to attain self-transcendence, they can optimize their physical and mental abilities and skills. This can lead to a sense of enjoyment and happiness derived from personal growth [45,46]. Given this conclusion, it is advisable for cyclists to prioritize the attainment of meaningful heart flow experiences through cycling rather than simply using it as a way to pass time.

Furthermore, the study has investigated the mechanism by which cyclists' recreational specialization indirectly impacts their heart flow experience through the mediating factor of environmental fit. This indicates that a higher level of recreational specialization among cyclists results in a better alignment between their skills, expertise, and the environment, leading to an enhanced sense of heart flow experience. Thus, the research confirms that individual-environment fit serves as a mediator between cyclists' recreational specialization and heart flow experience.

## 5 Research limitations

The study is focused on cyclists in Henan Province, which demonstrates regional characteristics, and the generalizability of the research findings is limited. The research solely examined the experiences of cycling enthusiasts during their rides using cross-sectional data to explore relationships among three potential variables. Therefore, it is recommended that future research be conducted over an extended period with a longitudinal design study focusing on cyclists to understand the developmental history of their recreational specialization and changes in the fit between individuals and their recreation environment and heart flow experiences. By analyzing the mutual influences of these variables over time more clearly, the research findings will have greater practical value.

## 6 Conclusion

The analysis has led to the development of a model illustrating the relationship between recreational specialization, individual-environment fit, and heart flow experience. Cyclists' level of recreational specialization directly and significantly impacts individual-environment fit. Individual-environment fit has a direct and significant positive effect on heart flow experience. Individual-environment fit acts as an intermediary variable between recreational specialization and heart flow experience, with a significant path. Recreational specialization directly and significantly influences heart flow experience. This suggests that high levels of recreational specialization among cyclists must be in line with the cycling environment in order to generate a high degree of heart flow experience. Cyclists are encouraged to focus on cultivating a cohesive team emotional atmosphere and enhancing their sense of heart flow experience. It is also recommended that the government improve bicycle-only paths and cycling facilities that meet cycling standards, as well as establish a cycling service system to promote the development of regional cycling sports. Additionally, cyclists should systematically enhance their cycling skills, tackle routes of varying difficulties, and further develop their sense of heart flow experience.

## Funding

The fund was provided by the General Project of Humanities and Social Sciences Research of 10.13039/501100009101Henan Provincial Department of Education (No.2023-ZZJH-280); 2023 10.13039/501100011447Henan Provincial Department of Science and Technology Soft Science Project (No.232400411107).

## Ethics approval and consent to participate

The research project does not include any animal experiments or human drug experiments. It is a public welfare project, and the informed consent is obtained before the research. After discussion by the ethics committee of College of physical education, Henan University of science and technology, the project research was approved (Project No: 20231002). Before participating in the study, each participant's written informed consent was solicited.

## Data availability

Supplementary material for this article is available online(weiye4488@163.com). Data online Nutstor: https://www. jianguoyun. com/d/home#/

## CRediT authorship contribution statement

**Wei Ye:** Writing – review & editing, Writing – original draft, Visualization, Validation, Supervision, Software, Resources, Project administration, Methodology, Investigation, Funding acquisition, Formal analysis, Data curation, Conceptualization.

## Declaration of competing interest

The authors declare the following financial interests/personal relationships which may be considered as potential competing interests:WEI Ye reports financial support was provided by 10.13039/501100009101Henan Provincial Department of Education and 10.13039/501100011447Henan Provincial Department of Science and Technology. If there are other authors, they declare that he has no known competing financial interests or personal relationships that could have appeared to influence the work reported in this paper.
