# Supplementary material for: An empirical study on the relationship between rider recreational specialization, individual-environment fit, and heart flow experience
Source: Heliyon. 2024 May 23;10(11):e31781. doi: 10.1016/j.heliyon.2024.e31781 (PMC11140799; doi:10.1016/j.heliyon.2024.e31781)
Supplement: Multimedia component 1 [file mmc1.docx]

**An Empirical Study on the Relationship between Rider Recreational Specialization, Individual-Environment Fit, and Heart Flow Experience（ALL-1）**

Hello!

The research is a scientific research project. Please fill in according to the researcher's explanation Please mark the appropriate option with a √.

All scales are measured using a Likert five-point scale (1-5 scores), which are represented as "strongly disagree", "disagree", "not sure", "agree", and "strongly agree".

**Researchers explain the purpose and content of the study and fill in the rules.**

1. Age; ( ≤30 31-45 ≥46 ) ;

2. Gender (  male female );

3. Participation years (0.6-1 1-2（inclusive） ＞2 )；

4. Educational level（ Junior college education or below Bachelor's degree or above is required）

**5. Research questionnaire**

| structural plane | Serial number | Factors | Option(s) |
| --- | --- | --- | --- |
| Specialization of Recreation | 1 | Engaging in cycling can give me a sense of superiority. | 1, 2, 3, 4, 5 |
|  | 2 | Cycling plays an important role in my leisure life | 1, 2, 3, 4, 5 |
|  | 3 | I have many friends who are involved in cycling activities | 1, 2, 3, 4, 5 |
|  | 4 | It's a very happy thing to participate in cycling | 1, 2, 3, 4, 5 |
|  | 5 | I often pay attention to (collect) cycling-related information | 1, 2, 3, 4, 5 |
|  | 6 | I am willing to invest in cycling activities (hardware and software) | 1, 2, 3, 4, 5 |
|  | 7 | I try to participate in my partner's cycling as much as possible | 1, 2, 3, 4, 5 |
|  | 8 | My leisure activities mostly focus on cycling activities | 1, 2, 3, 4, 5 |
|  | 9 | I like to challenge the road environment | 1, 2, 3, 4, 5 |
|  | 10 | I often discuss cycling experience with my companions | 1, 2, 3, 4, 5 |
|  | 11 | I have the knowledge or ability to participate in cycling activities | 1, 2, 3, 4, 5 |
|  | 12 | My riding ability can handle different Cycling field | 1, 2, 3, 4, 5 |
|  | 13 | My riding ability is sufficient to cope with any situation that may occur during the activity | 1, 2, 3, 4, 5 |
|  | 14 | Have predictive understanding of the cycling environment based on their own cycling experience (or information collection). | 1, 2, 3, 4, 5 |
|  | 15 | I have the ability to compare with other cyclists | 1, 2, 3, 4, 5 |
| **Individual-Environment Fit** | 16 | The relevant knowledge of cycling that I have is sufficient for me to fully utilize it in this environment. | 1, 2, 3, 4, 5 |
|  | 17 | I have enough mountain biking experience to ride in this environment. | 1, 2, 3, 4, 5 |
|  | 18 | The mountain biking skills I have are enough for me to fully utilize in this environment. | 1, 2, 3, 4, 5 |
|  | 19 | For cycling in specific environments, I will explore (seek advice from) new cycling skills. | 1, 2, 3, 4, 5 |
|  | 20 | This environment can make me happy | 1, 2, 3, 4, 5 |
|  | 21 | This environment makes me feel comfortable and relaxed when I ride | 1, 2, 3, 4, 5 |
|  | 22 | This environment can meet my expectations of the riding experience | 1, 2, 3, 4, 5 |
|  | 23 | This environment makes me feel challenging during the ride | 1, 2, 3, 4, 5 |
|  | 24 | My cycling tools meet my cycling needs in this environment | 1, 2, 3, 4, 5 |
|  | 25 | My cycling equipment meets my cycling needs in this environment | 1, 2, 3, 4, 5 |
|  | 26 | This environment allows me to share my experience with other cyclists. | 1, 2, 3, 4, 5 |
|  | 27 | This environment provides me with opportunities to meet other cyclists. | 1, 2, 3, 4, 5 |
|  | 28 | Interacting with other cyclists in this environment can improve my cycling skills. | 1, 2, 3, 4, 5 |
|  | 29 | The topographical characteristics of this environment are suitable for my cycling activities. | 1, 2, 3, 4, 5 |
|  | 30 | The resource conditions of this environment are suitable for me to engage in cycling activities. | 1, 2, 3, 4, 5 |
|  | 31 | The facilities (such as road signs) in this environment meet my expectations for cycling. | 1, 2, 3, 4, 5 |
|  | 32 | The convenience of transportation in this environment is suitable for me to engage in cycling activities. | 1, 2, 3, 4, 5 |
| **Flow Experience** | 33 | When riding, I feel that I can cope with sudden changes. | 1, 2, 3, 4, 5 |
|  | 34 | When I ride, I feel like I am in control of everything. | 1, 2, 3, 4, 5 |
|  | 35 | Cycling allows me to think of nothing else | 1, 2, 3, 4, 5 |
|  | 36 | When I ride, I will be fully focused on the ride. | 1, 2, 3, 4, 5 |
|  | 37 | Cycling gives me a pleasant experience | 1, 2, 3, 4, 5 |
|  | 38 | Cycling allows me to fully express myself | 1, 2, 3, 4, 5 |
|  | 39 | Cycling gives me a sense of exercise benefit (educational, physical and mental, social benefit) | 1, 2, 3, 4, 5 |
|  | 40 | When riding, I don't need to think hard to make the right action. | 1, 2, 3, 4, 5 |
|  | 41 | When riding, things seem to happen naturally. | 1, 2, 3, 4, 5 |
|  | 42 | When riding, time seems to pass quickly. | 1, 2, 3, 4, 5 |
|  | 43 | When riding, I don't pay attention to the existence of time | 1, 2, 3, 4, 5 |
|  | 44 | During the ride, I knew very clearly what I had to do | 1, 2, 3, 4, 5 |
|  | 45 | During the ride, my goal is clearly defined | 1, 2, 3, 4, 5 |
|  | 46 | Cycling can give me a sense of achievement | 1, 2, 3, 4, 5 |
|  | 47 | Cycling can give me a sense of challenge | 1, 2, 3, 4, 5 |
